# Anti-Inflammatory and Immunomodulatory Mechanism of Tanshinone IIA for Atherosclerosis

**DOI:** 10.1155/2014/267976

**Published:** 2014-12-02

**Authors:** Zhuo Chen, Hao Xu

**Affiliations:** ^1^Graduate School, Beijing University of Chinese Medicine, Beijing 100029, China; ^2^Cardiovascular Diseases Center, Xiyuan Hospital, China Academy of Chinese Medical Sciences, Beijing 100091, China

## Abstract

Tanshinone IIA (Tan II A) is widely used in the treatment of cardiovascular diseases as an active component of *Salvia miltiorrhiza* Bunge. It has been demonstrated to have pleiotropic effects for atherosclerosis. From the anti-inflammatory and immunomodulatory mechanism perspective, this paper reviewed major progresses of Tan IIA in antiatherosclerosis research, including immune cells, antigens, cytokines, and cell signaling pathways.

## 1. Introduction

When talking about the pathogenesis of atherosclerosis (AS), there has been a variety of hypotheses, such as thrombogenic theory, lipid infiltration theory, monoclonal hypothesis, response-to-injury hypothesis, oxidative hypothesis, stem cells hypothesis on atherogenesis, homocysteine theory, arginine hypothesis, shear stress hypothesis, and smooth muscle mutation theory. However, we could not completely explain the pathogenesis of AS yet. In the early 1990s, Dr. Ross proposed chronic inflammation hypothesis of AS. He further clearly regarded AS as an inflammatory disease in 1999 [1]. With more and more inflammatory cells and mediators constantly being detected, AS is no longer considered to be a simple disease that lipid deposited onto the arterial wall, but a progressive inflammatory response within the vessel wall. On the other hand, both innate and acquired immune responses mediate all stages of AS inflammation from initiation through progression [2, 3]; thus, immunomodulatory mechanism is also increasingly becoming a research hotspot [4].

*Salvia miltiorrhiza* Bunge is first recorded in the* Shen Nong's Herbal Classic*. It is the root of* Salvia miltiorrhiza* of Labiatae, bitter in taste, and a little cold in property, entering to the heart, pericardium, and kidney meridians. The action of* Salvia miltiorrhiza* is promoting blood circulation, regulating menstruation, removing blood stasis, relieving pain, cooling blood, eliminating carbuncle, and tranquilization. Its main chemical compositions can be divided into two categories: lipophilic diterpene quinones and water-soluble phenolic acids. Tanshinone IIA (Tan IIA) is one of the lipophilic constituents. Due to poor intestinal absorption and slow action onset of Tan IIA, Chien et al. [5] developed sodium Tan IIA sulfonate (STS) to improve bioavailability of Tan IIA. STS is researched in a variety of experiments and clinical studies thereafter. Tan IIA was found to have diverse pharmacological effects such as antioxidant, antibacterial, anti-inflammatory, hormone-like, and neuroprotective effects, and it has been used to treat cardiovascular disease, tumor, diabetes, liver disease, and so forth [6–9]. Especially, Tan IIA is even regarded as a promising natural cardioprotective agent [10]. For example, Tan IIA can not only dilate coronary artery, lower blood pressure and lipid, inhibit left ventricular hypertrophy, inhibit smooth muscle cell proliferation and intimal hyperplasia, reverse myocardial hypertrophy, reduce infarct size, and protect from ischemia-reperfusion injury but also has anti-AS, anti-inflammatory, antioxidant, antiplatelet, anticoagulant, antithrombotic, antiarrhythmic, and antimyocardial ischemia effects [11–14]. Among all of these effects, the anti-inflammatory and immunomodulatory mechanism one for AS has been highlighted in recent years.

## 2. Anti-Inflammatory and Immunomodulatory Mechanism of Tan IIA for AS

### 2.1. The Impact of Tan IIA on Immune Cells

#### 2.1.1. Dendritic Cells

Dendritic cells (DC), the most potent full-time antigen-presenting cells (APC), can present antigen to thymus dependent lymphocytes (T-lymphocytes) and induce innate immune responses. DC are derived from CD34+ hematopoietic progenitor cells in bone marrow. There are three differentiation stages: precursors, immature cells, and mature cells. DC are incapable of activating T-lymphocytes in stage of precursors and immature cells. During the process of upregulation of expression of adhesion molecules (CD54, CD58, CD11a/CD18, and CD50), costimulatory molecules (CD40, CD80, and CD86) and antigen-presenting molecules (MHCI, MHC II, and CD1) and the process of weakening of both endocytosis and antigen processing ability, DC become mature gradually. In stage of mature DC, the capacity of antigen presentation to T-lymphocytes is greatly strengthened.

Li et al. [15] found that Tan IIA could dose dependently downregulate expression of costimulatory molecules (CD86), adhesion molecules (CD54), and major histocompatibility complex (human leukocyte antigen-DR), restore the capacity for endocytosis, prevent DC from maturation, and reduce the secretion of proinflammatory cytokines interleukin- (IL-) 12 and IL-1 by human DCs. Hence, Tan IIA might attenuate the growth of atherosclerotic lesions via inhibiting dendritic cell-mediated adaptive immunity.

#### 2.1.2. Macrophages/Monocytes

Macrophages take in a large number of oxidized low density lipoproteins (oxLDL) under the intima and then become foam cells, which is one of the early signs of atherosclerotic lesions. ATP-binding cassette transporter A1 (ABCA1) is an integral membrane protein that transports lipid from the cells to the liver with subsequent lipid excretion by bile at last. This reversal transport inhibits the formation of lipid. Hu et al. [16] found that Tan IIA significantly increased expression of ABCA1 in macrophage-derived foam cells, raised the outflow of intracellular cholesterol, and inhibited the formation of oxLDL-induced foam cells. Furthermore, the liver X receptor (LXR) and peroxisome proliferator activated receptor (PPAR) which are members of nuclear receptor superfamily can increase expression of ABCA1 through coupling path. Lu et al. [17] also proved Tan IIA inhibited the formation of foam cells. The mechanism might be that Tan IIA increased expression of PPAR*α*, LXR*α*, and ABCA1, facilitated reversal cholesterol transport, and ultimately promoted cholesterol efflux.

#### 2.1.3. T-Lymphocytes

T-lymphocytes in plaques are mostly CD4+ T-lymphocytes which belong to Th1 subtype. Most of CD4+ T-lymphocytes are active and can induce “cytokine waterfall” as well as secrete proinflammatory cytokines interferon-gamma (IFN-*γ*), IL-12, and other cytokines, eventually leading to inflammation. Experiments showed that tanshinone significantly inhibited production of Th1-derived IL-12 and IFN-*γ* in a dose-dependent manner [18]. T-lymphocytes play a role in the formation of AS, mainly because of proinflammatory cytokines secreted by T helper type (Th) 1 or Th2. Therefore, tanshinone can inhibit the formation of AS and stabilize plaques via reducing the secretion of Th1 or Th2. Moreover, T-lymphocytes are activated to express CD40L, and the expression of tissue factor and matrix metalloproteinases (MMPs) is then promoted by interaction between CD40L and CD40. Fang et al. found that Tan IIA could improve the activity of superoxide dismutase (SOD), decrease the level of malondialdehyde (MDA), and reduce both the expression of CD40 and the activity of MMP-2. The antioxidation and anti-inflammation effect of Tan IIA might be one of its potential mechanisms in treating AS [19]. Lin et al. [20] indicated that Tan IIA could protect a human umbilical vein endothelial cell (HUVEC) line (ECV-304) damage induced by hydrogen peroxide by means of antioxidation and anti-inflammation, and CD40 expression was decreased in a dose-dependent manner in vitro.

### 2.2. The Impact of Tan IIA on Antigen

#### 2.2.1. OxLDL

OxLDL is an antigen which can lead to AS. OxLDL antibodies can be detected in serum or plaque of both healthy people and patients suffering from AS. OxLDL can injure endothelial cells; this cytotoxicity is embodied in increased expression of intercellular adhesion molecule (ICAM-1), vascular cell adhesion molecule-1 (VCAM-1), and other inflammatory factors in endothelial cells which induce endothelial adhesion. Inflammation can further activate MMPs to degrade extracellular matrix, which weaken the stability of fibrous cap. As a result, stable plaques change into vulnerable ones.

*Salvia miltiorrhiza* plays a role in endothelium protection and plaque stabilization since it can weaken the impact of oxLDL stimulating endothelial cells to express ICAM-1 and MMP-9 [21]. OxLDL induces the formation of foam cells, monocytes adhesion to endothelial cells, and aggregation of platelets and thrombosis. Therefore, oxLDL plays a vital role in the formation of AS. Both in vivo [22, 23] and in vitro experiments [23] demonstrated that Tan IIA reduced the oxLDL production.

#### 2.2.2. Heat Shock Proteins

Heat shock proteins (HSP) are known to enhance cells' ability to survive life-threatening stress and have immunogenic properties. The interaction of HSP with a variety of antigen peptides induces an effective specific antigen immune response. HSP70 was first discovered in plaques of human and rabbits suffering from AS by Berberian et al. [24]. In human bodies, HSP65/60 antibodies are increased in early stage of AS. The cells can respond to external stimuli by virtue of stress protein. Different types of HSP have different effects. For example, HSP60 as an autoantigen can cause cellular and humoral immunity, while HSP70 plays a cytoprotective role. Zhou et al. [25] found that Tan IIA significantly inhibited superoxide production and expression of NADPH oxidase (NOX4) and increased NO production, endothelial nitric oxide synthase (eNOS) homodimerization, HSP90, GTPCH1, and DHFR in a concentration-dependent manner. Tan IIA enabled cells to restore eNOS coupling, which resulted in reduced intracellular oxidative stress and increased NO production.

### 2.3. The Impact of Tan IIA on Cytokines

Cytokines can be classified according to their characters whether proinflammatory or anti-inflammatory. Proinflammatory cytokines include tumor necrosis factor (TNF), IL-1, IL-12, IL-18, and IFN-*γ*. Anti-inflammatory cytokines include IL-4, IL-10, IL-13, and transforming growth factor-*β* (TGF-*β*). Most of proinflammatory cytokines can cause AS, while anti-inflammatory cytokines show different effects on atherosclerosis. TGF-*β* and IL-10 are beneficial in preventing AS. On the contrary, anti-inflammatory cytokines IL-4 and IL-13 might have atherogenic effect. IL-6 is a pleiotropic cytokine of a dual role, which can be both anti-inflammatory and proinflammatory. Experiments showed that Tan IIA increased mRNA expression of anti-inflammatory cytokine IL-10 [26, 27] and reduced expression of IL-6 in RAW264.7 cells pretreated with LPS [27].

VCAM-1 is only expressed when there is inflammation or other stimuli in vascular endothelium. In the early stage of AS, VCAM-1 promotes adhesion and metastasis of leukocytes and macrophages, induces macrophages to uptake lipid and change themselves into foam cells. and promotes lymphocyte adhesion to endothelial cells. In the late stage of AS, VCAM-1 intensifies local inflammation and results in macrophage accumulation, eventually inducing secretion of MMP which can degrade the fibrous cap. Li et al. [28] showed Tan IIA inhibited atherosclerotic progression, which might be related to the decreased expression of VCAM-1. Another in vitro experiment [29] manifested Tan IIA inhibited the expression of adhesion molecules (ICAM-1, P-selectin) in HUVEC and platelets in a concentration-dependent manner.

IL-8 and monocyte chemoattractant protein-1 (MCP-1) are both chemokines which induce monocyte adhesion to endothelial cells. IL-8 may attract T-lymphocytes and bring about proliferation and migration of smooth muscle cells (SMC). Meanwhile, MCP-1 can induce local infiltration, collection, and proliferation of monocytes/macrophages. Zhang et al. [30] found that Tan IIA significantly reduced not only the serum concentration of IL-8 in AS rabbits but also the expression of VCAM-1 in AS rabbits' aorta. Yi indicated that Tan IIA decreased the protein expression of Bax in AS plaques and mildly increased that of Bcl-2, which resulted in reduced ratio of Bax to Bcl-2 as well as the expression of MCP-1, thus concluding that Tan IIA inhibited atherogenesis by means of regulating the apoptosis and expression of inflammatory factors [31].

MMP can degrade extracellular matrix, which induces further degradation of the fibrous cap and ultimately results in plaque rupture. Among all subtypes, type IV collagenases (MMP-2 and MMP-9) participate in local inflammatory cell infiltration in plaques and result in damage to the vessel wall and intimal defense function decline. MMP-9 promotes SMC to migrate to the intima and accelerate AS lesions. Xu et al. [32] showed that Tan IIA reduced lesion size in the aortic sinus in ApoE mice, made plaques more stable, reduced macrophage infiltration, and increased SMC and collagen contents. Tan IIA significantly decreased in situ superoxide anion production, aorta expression of nuclear factor-*κ*B (NF-*κ*B), MMP-9, and other proinflammatory cytokines (IL-6, TNF-*α*, MCP-1). Through reducing vascular oxidative stress and inflammatory response, Tan IIA attenuates the development of AS lesions.

It was also reported that Tan IIA inhibited aortic atherosclerotic lesions formation and induced a reduction of protein expression and activities of MMP-2 and MMP-9 as well as serum VCAM-1 and IL-1*β* in high-fat fed rabbits in a dose-dependent manner [33].

### 2.4. The Impact of Tan IIA on Cell Signaling Pathways

#### 2.4.1. Toll-Like Receptors/NF-*κ*B Pathways

Toll-like receptors (TLRs) are bridges that connect innate immunity and acquired immunity. When TLRs are activated, signaling pathways will be divided into MyD88-dependent and non-MyD88-dependent ones. The key link of the former one is NF-*κ*B. Once activated, NF-*κ*B can regulate the expressions of multiple inflammatory cytokines, such as MCP, and cell adhesion molecule (e.g., ICAM-1, VCAM-1, E-selectin, and P-selectin) so as to affect the AS process. Jia et al. [34, 35] demonstrated Tan IIA inhibited the mRNA and protein expression of TLR4, NF-*κ*B, and TNF-*α* in cultured HUVEC EA.hy926 induced by LPS. TNF-*α*, a downstream signal of TLR4/NF-*κ*B pathway, is in the central part of the inflammatory cascade of signaling pathways. The expression level of TNF-*α* may reflect the severity of inflammation. Huang et al. [36] found that Tan IIA inhibited AS mainly by blocking TLR4's identification of LPS in acute inflammation. Tan IIA prevented inflammatory signals from being transmitted into cells and then inhibited activity of NF-*κ*B and ultimately exerted anti-inflammatory effect.

Activation of NF-*κ*B, as one of the initiating mechanisms of endothelial cell damage, upregulates the expression of adhesion molecules in endothelial cells, thereby promoting monocyte adhesion to endothelial cells and subcutaneous inward migration. There is expression of ICAM-1 in both endothelial cells and SMC in AS plaques. Expression of ICAM-1 makes leukocyte infiltrate gather in certain parts, causing local tissue damage and inflammation. VCAM-1 mediates monocyte adhesion to endothelial cells by the integrin receptors, activates monocytes to transform into macrophages under endothelium and further into foam cells, and accelerates the formation of AS. Antigen expression of ICAM-1 and VCAM-1 may be a costimulatory signal of T-lymphocytes, further inducing lymphocyte's secretion of inflammatory cytokines. Tan IIA can inhibit the mRNA expression of ICAM-1 and VCAM-1 induced by the NF-*κ*B activation [37]. Chang et al. [38] confirmed that Tan IIA could modulate expression of VCAM-1, ICAM-1, and chemokine to exert inhibitory effect on AS through inhibition of TNF-*α*-induced activation of I-kappaB kinase (IKK)/NF-*κ*B signaling pathway in HUVEC. Wang and Shen [39] showed that Tan IIA significantly reduced the expression of NF-*κ*B p65, suggesting that Tan IIA protected vascular endothelial cells by inhibiting p65 expression of NF-*κ*B pathway to suppress inflammatory cascade.

#### 2.4.2. Mitogen-Activated Protein Kinase Pathway

Mitogen-activated protein kinases (MAPKs) are an important signal transduction pathway that regulates cell growth and apoptosis. MAPKs consist of three subfamilies: extracellular regulated protein kinases (ERKs), c-Jun N-terminal protein kinase (JNKs), and p38 protein kinase (p38 kinase). ERKs signaling pathway plays a major role in cell proliferation and is also involved in cell differentiation. On the contrary, JNKs and p38 pathways are closely related with apoptosis. Jang et al. [40] showed that Tan IIA inhibited LPS-induced IkappaB alpha (IKB*α*) degradation and NF-*κ*B activation by virtue of inhibition of NF-*κ*B induced kinase-I*κ*B kinase (NIK-IKK) pathway and MAPKs (p38, ERK1/2, and JNKs) pathways in RAW 264.7 cells.

#### 2.4.3. PPAR*γ* Pathway

Activation of PPAR*γ* can inhibit the expression of inflammatory chemokines, especially the expression of TNF-*α*, IL-1*β*, IL-6, nitric oxide synthase (NOS), and scavenger receptor A. Yi et al. [41] showed that Tan IIA significantly increased collagen content, PPAR*γ* protein, and IL-10 mRNA and reduced IL-6 mRNA and MMP-1 protein, thus stabilizing AS plaque via anti-inflammatory mechanism.

#### 2.4.4. Other Pathways

Tan IIA could selectively inhibit TNF-*α* mediated expression of VCAM-1 rather than ICAM-1, through regulation of the phosphatidylinositol-3kinase (PI3K)/Akt, protein kinase C (PKC), and Jak/STAT-3 pathway as well as binding activity of interferon regulatory factor (IRF-1) and GATA-6, which might be involved in the anti-AS mechanism of Tan IIA [42].

## 3. Final Comments

Researches on AS immune and inflammatory mechanism have attracted more and more attentions. The relevant therapeutic researches have also become hotspots in recent years [43]. Tan IIA is a promising natural cardioprotective agent with pleiotropic effects. It is found that Tan IIA can protect endothelial cells, prevent formation of foam cells, reduce expression of inflammatory cytokines, and intervene with multiple AS links through different pathways. Although many mechanisms have been preliminarily illuminated, there are still many questions left to be answered. For example, why can Tan IIA regulate expression of various cytokines? Is that caused by the effect of Tan IIA on a particular gene or protein in the upstream consequently affecting downstream cytokines? Or does Tan IIA itself has multiple targets? There is no doubt that future direction of research will combine various genomics technologies with miRNA research methods. Given the complexity of the pathogenesis of AS, in addition to applications of knockout animals in vivo studies, introduction of AS composite models due to a variety of risk factors is a good choice. Meanwhile, cell coculture system can be introduced into in vitro studies in order to further explore AS complex pathophysiological mechanisms and interactions between cells. Last but not least, we should further set specific blockers as a control group to offer higher quality evidence for the role of specific pathway in Tan IIA's intervention on AS. In short, the research on the immune and inflammatory mechanism of Tan IIA for AS is promising but still has lots of unknown areas to be explored.
